# Smiling doctor, satisfied patient—the impact of facial expressions on doctor-patient interactions

**DOI:** 10.3389/fmed.2025.1518517

**Published:** 2025-04-24

**Authors:** Pia Schneider, Giulia Zerbini, Philipp Reicherts, Miriam Reicherts, Nina Roob, Tobias Hallmen, Elisabeth André, Thomas Rotthoff, Miriam Kunz

**Affiliations:** ^1^Department of Medical Psychology and Sociology, Institute of Theoretical Medicine, Faculty of Medicine, University of Augsburg, Augsburg, Germany; ^2^Medical Education Sciences, DEMEDA, Faculty of Medicine, University of Augsburg, Augsburg, Germany; ^3^Department of Human-Centered Artificial Intelligence, Faculty of Applied Computer Science, University of Augsburg, Augsburg, Germany; ^4^Medical Didactics and Education Research, DEMEDA, Faculty of Medicine, University of Augsburg, Augsburg, Germany

**Keywords:** doctor-patient communication, facial expression, non-verbal communication, patient satisfaction, medical students

## Abstract

**Introduction:**

Although the importance of facial expressions for good doctor-patient communication is widely acknowledged, empirical evidence supporting this notion is scarce. We used a fine-grained, anatomically-based measure to investigate which facial expressions are displayed in (simulated) doctor-patient consultations and whether these can predict communication quality.

**Methods:**

Fifty two medical students engaged in simulated doctor-patient consultations with standardized patients (SPs) and their facial expressions were analyzed using the Facial-Action-Coding-System (FACS). The quality of the communication was rated by SPs, medical students, and by communication experts. SPs also rated their level of comfort.

**Results:**

The predominant facial expression being displayed by medical students was smiling. Medical students' smiling positively predicted the communication quality and level of comfort experienced by SPs. In contrast, smiling had little effect on medical students' self- and expert-assessments of communication quality. Smiling of medical students significantly predicted patient level of comfort and perceived quality of communication. This predictive power was found for genuine and for social smiles as well as for smiles displayed during speaking and during listening.

**Discussion:**

Smiling seems to be a robust non-verbal behavior that has the potential to improve doctor-patient communication. This knowledge should be taken into consideration in medical training programs.

## 1 Introduction

The significance of effective communication between medical practitioners and patients has gained increasing recognition in the past decades (1–5). Patients who perceive the communication as good and trustworthy exhibit higher adherence to treatment (6), remain with the same doctor for longer (7), experience fewer complications (8), and have better health outcomes (9). Given this importance, training of communication skills has been implemented in medical curricula in recent years (10–13). Most communication curricula focus on teaching both verbal (e.g., verbal mirroring, use of plain language) as well as nonverbal skills [e.g., body posture, eye contact (14–18)]. According to Argyle non-verbal behavior serves the purpose to express emotions, interpersonal attitudes and one's personality which helps to establish and maintain interpersonal relations (19). Despite the common understanding that nonverbal aspects also play an important role in doctor-patient communication (5, 20), empirical evidence supporting this notion is rather scarce. For example, in a recent review article on empathetic behaviors in doctor-patient communication, non-verbal behaviors were not considered in the included studies (21). There is only a handful of studies investigating the effect of nonverbal behavior on communication outcomes, which found that physicians who keep open body positions, maintain symmetrical arm postures and establish eye contact, are perceived as more empathic and competent (16, 22, 23).

Regarding the most prominent nonverbal behavior, namely facial expressions, evidence also points toward a significant association with patient satisfaction (15, 18, 24). Here, especially smiling seems to be relevant (24–27). Typically, facial expressions of the doctor or medical student (during simulated or real consultations) are assessed rather holistically using one-dimensional observer ratings of “facial expressiveness,” or of individual expressions, such as smiling (15, 17, 18, 28). For instance, one study rated medical students' facial expressions as either “bad” or “good” (28), whereas in another study facial expressivity and the frequency of smiling were rated on 7-point scales (17). Thus, although previous studies point toward an association between facial expressions (especially smiling) and communication outcomes, this evidence is not based on thorough, fine-grained, anatomically-based measures of facial expressions, such as the Facial Action Coding System (FACS) (29). The FACS measures visually distinguishable facial movements, which are called Action Units (AUs). FACS has the advantage of allowing an objective, detailed and comprehensive description of facial behavior that is less prone to subjective interpretation compared to other systems (30) such as the Maximally discriminative facial movement coding system (31).

To fill this gap, we used the FACS as a thorough methodology approach to investigate the following research questions, namely

(i) which facial expressions are displayed in doctor-patient consultations and
(ii) which facial expressions play an important role in predicting the outcome of doctor-patient consultations.

To accomplish this objective, medical students engaged in a 7-min consultation with a standardized patient (SP). Our study offers thus a significant contribution beyond previous research. Firstly, the context of the conversation could be held constant, unlike in real-world appointments where topics, needs and questions vary between patients. Secondly, SPs underwent training how to rate the quality of conversation and level of comfort, in order to reduce variability between SPs. Lastly, the study was conducted in a specialized video laboratory equipped to capture high-resolution facial videos, thus allowing for detailed analysis of facial expressions. We used the Facial Action Coding System (FACS) by Ekman et al. (29), which is recognized as the gold standard to analyze facial expressions (32).

We hypothesize that the facial expressions displayed by the medical students during the consultations impact the quality of the communication as well as the patients‘ level of comfort. Especially, facial expressions of positive emotions [e.g., AU 12 (zygomaticus major)] should predict better communication outcomes.

## 2 Materials and methods

### 2.1 Participants

A total of 52 medical students from the third semester of the medical faculty at the University of Augsburg (Germany) participated in the study. We used G^*^Power (33) to estimate the sample size. Assuming a medium-to-large effect size (f^2^ = 0.25), a significance level of α = 0.05 and a power of 0.80, resulted in a sample size of 42 participants. Given that some individuals are stoic in their facial expressions, we increased the sample size to 52 to ensure enough variability in facial expressiveness. The sample included 40 female and 12 male participants (mean age: 21.96 years, SD: 3.08). Thus, we have a clear gender bias in our sample, with mostly female participants, which could affect the outcome. However, the sex distribution of the participants did not differ significantly from the sex distribution of the corresponding semester (Chi^2^ = 2.79, *p* = 0.095), showing that this sex imbalance is a true representation of the sampled population of medical students. The sex distribution of the participants did not differ significantly from the sex distribution of the corresponding semester (Chi^2^ = 2.79, *p* = 0.095). Students received study credits for their participation. All participants were informed of how recordings will be used (FACS coding of their expression) and stored (on a secure server with restricted access) and provided written informed consent. The study protocol was approved by the ethics committee of the University of Bamberg.

### 2.2 Materials and procedure

**Figure 1:**
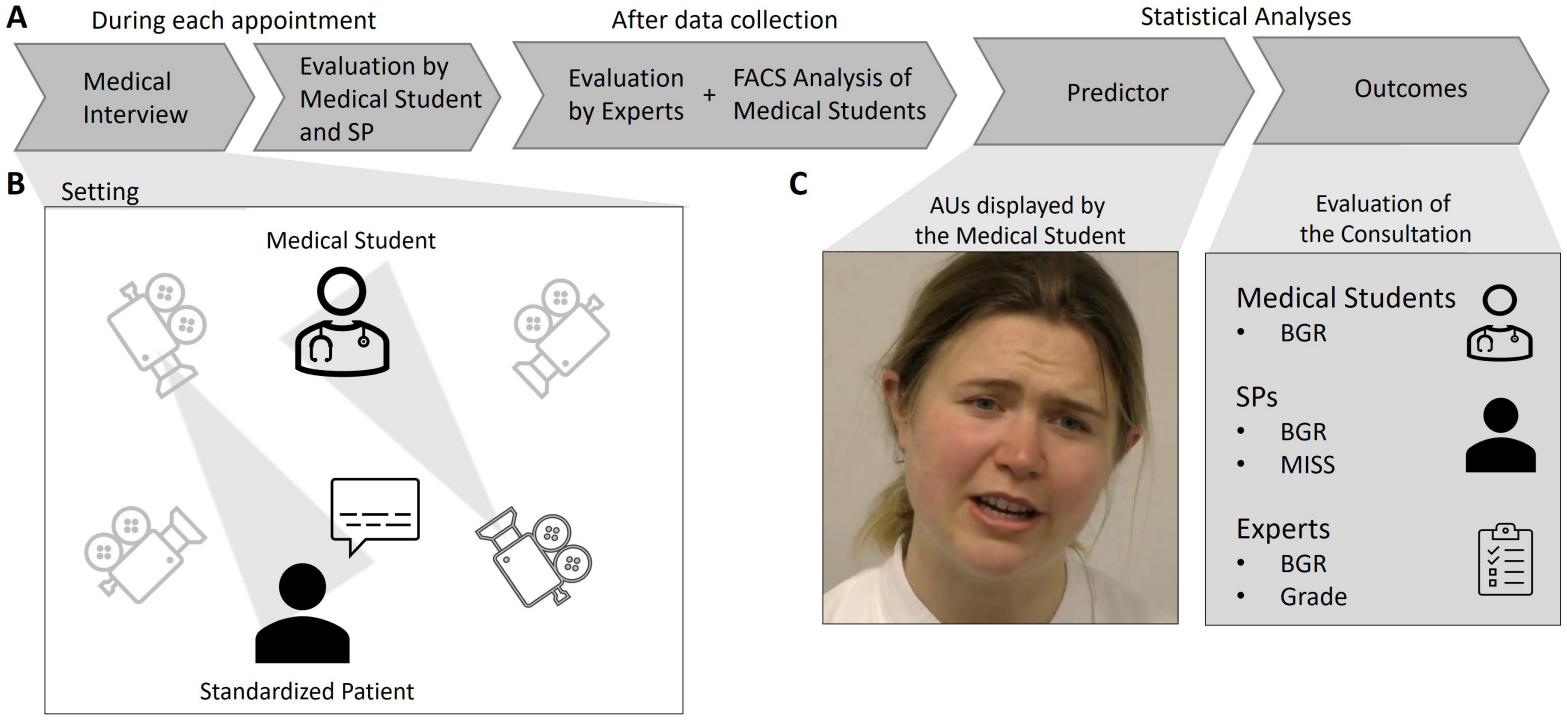
Overview of the study **(A)** and the data collection setup **(B)** as well as the predictor and outcome variables for the statistical analyses **(C)**. Only the medical consultation [setting depicted in **(B)**] was recorded on video. SP, Standardized Patient; FACS, Facial Action Coding System; AU, Action Unit; BGR, Berlin Global Rating; MISS, Medical Interview Satisfaction Scale.

The study was conducted in a laboratory equipped with modern video technology suitable for recording conversations and analyzing them offline. A general overview over the study procedure is shown in Figure 1.

After signing the consent form, medical students were asked to engage in a simulated medical consultation lasting approximately 7 min with a SP. The selection process of SPs involved an interview and two basic training workshops. SPs (*N* = 5) were selected based on good communication skills, their ability to provide constructive feedback, ability to take directions and their ability to memorize scripts and respond to different scenarios in role play exercises. They learned the case history, lifestyle, family background, personality, affective state and symptoms of the patient they are portraying based on a detailed case script. Moreover, non-verbal response patterns (e.g., body posture, facial expressions) and appropriate emotional responses were developed and practiced in order to maintain realism and standardization. As a last step, SPs practiced with a communication expert, to finetune the role. All SPs had acting experiences. The SPs received extensive training for their role. They played the role of a parent of a 3-month-old child who needed to be vaccinated. The SP acted as being slightly anxious about the vaccination and sought medical advice. In this scenario, the third semester medical students were instructed to apply the following communication techniques: conversation structuring and empathic communication (e.g., active listening, paraphrasing). Four high-resolution cameras (Panasonic, Model: AW-HE40S) were used to capture the face or the whole body of the medical student or the SP, respectively.

After the medical consultation, both the medical student and the SP independently evaluated the medical students' communication quality using standardized questionnaires described below. After data collection, four experts who are faculty members involved in teaching the communication curriculum (called communication experts in the remainder) watched the video recordings and evaluated the medical students' communication quality. Prior to this, all experts discussed the scoring criteria using example videos in a two-hour training session. Interrater reliability (Intraclass Correlation Coefficient, ICC) between experts was evaluated using 9 randomly selected videos which were rated by all four experts. We found good interrater reliability between experts (see exact values in Section 2.4.1).

### 2.3 Predictor

The student's face was recorded on video throughout the medical consultation. Facial responses were quantified using the Facial Action Coding System (FACS), a fine-grained anatomically based system that is considered the gold standard for facial expression analyses (34). The FACS is based on the anatomical analysis of facial movements and distinguishes 44 different Action Units (AUs) produced by a single muscle or a combination of muscles. FACS also incorporates AU 50 to indicate incidences when a person is speaking (29). A certified FACS coder identified the duration and intensity (measured on a 5-point scale) of the different AUs. The coder was qualified through successful completion of an examination administered by the system's developers. To calculate interrater reliability, 30 seconds of 20% of videos were additionally FACS coded and the Ekman-Friesen formula (35) was used to compute reliability, reaching 90.5% agreement between FACS codes, which compared favorably to previous studies (35). FACS coding was performed using specialized software, namely the Observer Video-Pro (Noldus Information Technology). Given the enormous time it takes to manually FACS code (it took approximately 20 h to FACS code one 7-min medical consultation), only the facial expressions of the medical students were FACS coded. The FACS coder was blind to the outcome variables (communication quality and level of comfort ratings). For further analyses, intensity and duration values of each AU will be multiplied (product term) and AUs will be averaged to form composite scores of facial expressions.

### 2.4 Outcomes

The simulated doctor-patient communication was evaluated using standardized questionnaires and rating scales (see below) that were filled out by the medical students, SPs and communication experts. Both the quality of the communication as well as the level of comfort felt by the SPs were assessed.

#### 2.4.1 Communication quality

##### 2.4.1.1 Berlin Global Rating (BGR)

The BGR scale was developed by Hodges and McIlroy (36) and has been validated in German by Scheffer, showing good psychometric properties (37). We chose the BGR because it is frequently used to evaluate the quality of doctor-patient communication, especially in educational settings (38, 39). Moreover, the BGR is easily applicable due to its simple structure: 4 aspects are measured, namely empathy, coherence, verbal, and non-verbal communication. Each aspect is assessed with one item on a 5-point Likert scale. At both ends of the scale, descriptions of positive and negative examples are provided. For instance, the positive example for non-verbal communication states: “The student consistently engages the patient through non-verbal expression or encourages their participation in the conversation.” Notably, specific non-verbal behaviors such as smiling are not evaluated. An average BGR score was computed across the 4 aspects.

The BGR was filled out by SPs, medical students as well as communication experts.

##### 2.4.1.2 Grade

In addition to the BGR, the communication experts graded the student's performance based on a grading scheme, ranging from 1.0 (best) to 5.0 (worst) in 0.3 increments. The students were expected to apply communication techniques such as conversation structuring, and empathic communication and the application of these techniques was graded.

Interrater reliability (Intraclass Correlation Coefficient, ICC) between experts (11 videos were rated by all four experts) showed moderate to good agreement between experts (ICC_grade_ = 0.85; ICC_BGR_ = 0.77) (40).

#### 2.4.2 Assessment of level of comfort (MISS)

The Medical Interview Satisfaction Scale (MISS-21) was used to assess SP's level of comfort during the medical consultation. We chose this scale because it is widely used to assess patient satisfaction in consultations and has shown good psychometric properties (reliability and validity) (41, 42). We used a shortened version with 7 items of the “rapport” and “communication comfort” subscales (items 6, 8, 9, 10, 11, 12, 14 of the MISS-21 were selected) which were more suitable for our simulated medical consultation (e. g., “The doctor gave me a chance to say what was really on my mind.”). Items that were not applicable to our simulated consultation (e.g., “I'm unsure whether the doctor's treatment will be worth the effort”) were therefore excluded. Each item was rated on a 5-point Likert scale.

### 2.5 Statistical analysis

#### 2.5.1 Description of facial expressions displayed by the medical students [research question (i)]

a. Repertoire of facial movements: In a first step, we wanted to find out which AUs are displayed more consistently by medical students in a patient-doctor consultation. To this aim, we selected only those AUs that were displayed on average for at least 10 seconds across the 7 min consultations (which equals approximately to 2.5% of consultation time) to filter-out less frequent and thus, less relevant AUs. The “2.5% threshold” was chosen as a very liberal threshold [other studies used 5% (43)] in order not to miss any AUs that might be of importance.
b. Facial expressions during speaking and listening: We used paired *t*-tests to investigate whether medical students' facial expressions differed depending on whether they were speaking or listening (44). This has not been previously analyzed in doctor-patient communication.

#### 2.5.2 Analysis of predictors of communication outcomes [research question (ii)]

Given that we assessed several outcome measures, we first examined correlations between outcome measures using Pearson correlation.

For our main hypothesis, namely if medical students' facial expressions can predict communication outcomes, we used linear regression analyses. Composite scores of facial expressions were entered as predictors and regression analyses were conducted separately for each outcome measure (see below). Sex was included as a dummy variable in all analyses to control for its potential effects.

a. Communication Quality: The mean BGR rating of each rater group (SPs, medical students, communication experts) served as outcome measure for three separate regression analyses. Furthermore, the grade assigned by the communication experts was used as another outcome measure for an additional regression analysis.
b. Level of comfort: Level of comfort was only assessed by SPs using MISS. Thus, one regression analysis was conducted, with SPs comfort levels as the outcome variable.

Statistical significance was defined as alpha < 0.05. Assumptions of non-collinearity and normality were checked and were acceptable for the regression models. R (version 4.1.0) using R studio (version 1.1.463) was used for the statistical analyses.

## 3 Results

### 3.1 Repertoire of facial movements being displayed by the medical students [research question (i)]

Supplementary Table S1 gives an overview on the duration of all 44 FACS Action Units. Out of all AUs, only eight AUs were displayed on average for at least 10 seconds across the 7 min medical consultation (see Table 1). Following the evidence from previous studies, three AUs reported in Table 1 constitute a “smile,” namely AUs 06, 07, and 12 (45, 46). Ekman et al. (47) distinguished between two types of smiles, a Duchenne and a non-Duchenne smile. Duchenne smiles (“genuine” or “felt” smiles), are characterized by the contraction of the zygomatic major muscles (AU 12), eliciting an elevation of the corners of the mouth, and the simultaneous contraction of the orbicularis oculi muscles (AUs 06, 07). Non-Duchenne smiles (social smiles) often only include the contraction of the zygomatic major muscles (AU 12). To provide further insight into the nature of the smile of the medical students, we analyzed how often AU 12 was displayed together with AUs 06 and/or AU 07. Our results show that in 50.42% of the time, AU 12 was accompanied by AU 06 and/or AU 07. This indicates that medical students displayed genuine as well as social smiles (with comparable occurrences) during the consultation.

**Table 1:** Facial expressions of the medical students: AUs displayed on average for at least 10 seconds across the 7 min consultation.

**Action Units**	**Description**	**Muscle**	**Mean duration (in seconds) ±SD**	**% of occurrence during the ~7 min. conversation**
AU 01 ^b^	Inner brow raiser	frontalis	22.04 ± 33.03	5.22
AU 02 ^b^	Outer brow raiser	frontalis	20.48 ± 30.95	4.85
AU 04 ^b^	Brow lowerer	corrugator supercilli	12.09 ± 17.59	2.86
AU 06 ^a^	Cheek raiser and lid compressor	orbicularis oculi	28.30 ± 64.30	6.70
AU 07 ^a^	Lid tightener	orbicularis oculi	85.05 ± 74.86	20.15
AU 12 ^a^	Lip corner puller	zygomatic major	116.13 ± 98.09	27.51
AU 25 ^b^	Lips part	orbicularis oris muscle	45.19 ± 31.81	10.71
AU 26 ^b^	Jaw drop	orbicularis oris muscle	35.02 ± 27.04	8.29

^a^We combined these AUs into a composite score of “smiling.” ^b^These AUs were combined into a composite score of “other facial expressions.”

**Figure 2:**
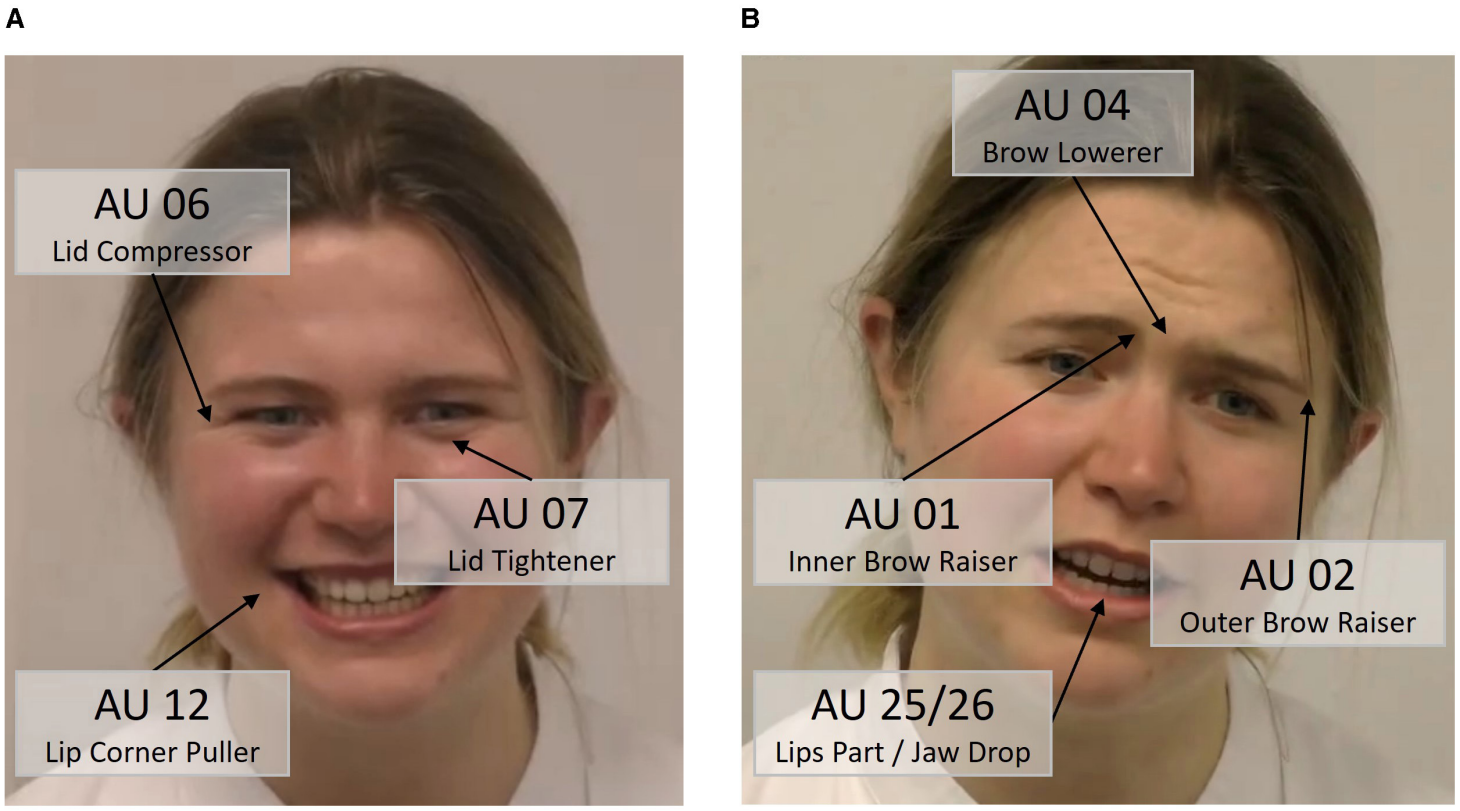
Examples of our extracted two categories of facial expressions displayed by the medical students. **(A)** Smiling, **(B)** other facial expressions.

For further analyses, we divided the AUs in Table 1 into two categories of facial expressions: a smiling expression (AUs 06, 07, 12) and a rest category of “other facial expressions” (AUs 01, 02, 04, 25, 26) (see examples in Figure 2). For those two categories of AUs composite scores were calculated by multiplying the intensity and duration values of each AU (creating a product term) and then averaging these product terms across the relevant AUs to form composite scores. This method provides a measure that accounts for the intensity and duration of multiple AUs. Since the data had strongly positively skewed distributions and heterogeneous variances, we transformed the composite scores using a square-root transformation (sqrt) (48).

#### 3.1.1 Facial expressions during speaking and listening

**Figure 3:**
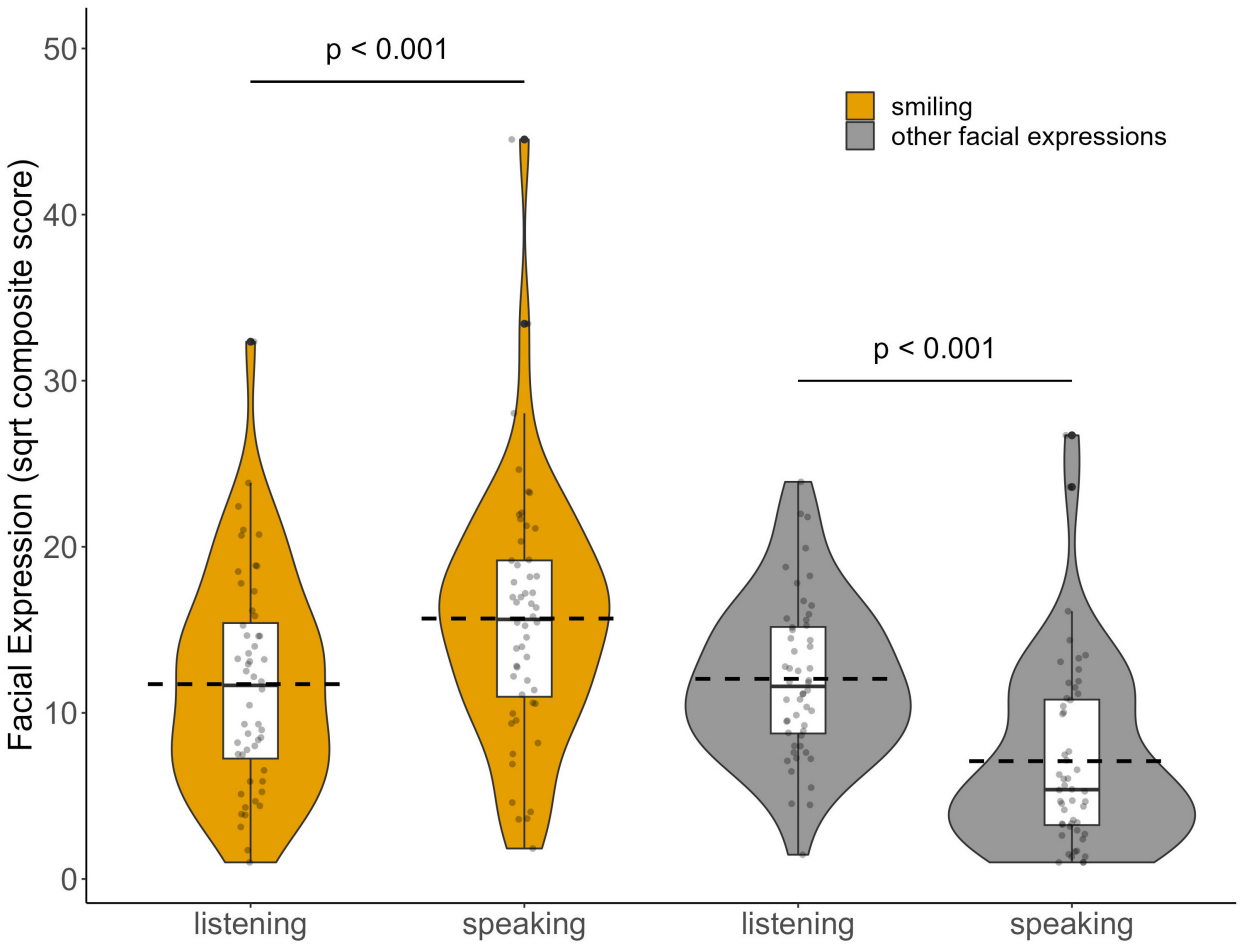
Medical students' facial expressions while listening and while speaking. Distribution of sqrt composite scores of smiling (AUs 06, 07 and 12) and other facial expressions (AUs 01, 02, 04, 25, and 26) are displayed as violin graphs, with the dashed horizontal lines indicating the mean. *p*-values represent statistical differences between speaking compared to listening intervals (paired *t*-tests).

Speaking times (AU 50) were approximately equal between SPs and medical students, with medical students speaking on average for 3:40 min (SD = 48 sec.) and SPs speaking on average for 3:18 min (SD = 33 sec.). Paired t-tests showed that medical students smiled significantly less when listening compared to speaking [t_(51)_ = −7.22; *p* < 0.001) but displayed significantly more other facial expressions when listening compared to speaking [t_(51)_ = 6.31; *p* < 0.001] as can be seen in Figure 3.

#### 3.1.2 Summary for research question (i)

In sum, we found that especially smiling was displayed frequently by medical students, with comparable displays of genuine and social smiles. Besides smiling, movements of the eyebrows as well as opening of the mouth formed the second category of facial expressions (other facial expressions). While smiling was displayed more frequently when the medical students were speaking, “other facial expressions” were more prominent during listening segments.

### 3.2 Prediction of communication outcomes [research question (ii)]

Given that we assessed multiple communication outcome measures from three different groups to evaluate “communication quality,” we first investigated correlations between the different outcome measures. We found significant correlations between BGR expert and BGR medical student ratings (r = 0.34, *p* = 0.01) as well as between BGR expert and BRG SPs ratings (r = 0.42, *p* < 0.01). In contrast, the students' and SPs' BGR ratings were not significantly correlated (r = 0.01, *p* = 0.93). The grade assigned by the experts correlated significantly with all three BGR-ratings (BGR experts: r = 0.91, *p* < 0.01; BGR students: r = 0.28, *p* = 0.04; BGR SPs: r = 0.47, *p* < 0.01).

To address our primary aim of examining the predictive potential of facial expressions on communication outcomes, we used three-stage hierarchical multiple regression analyses with the enter method (see Table 2). This approach allowed us to introduce variables at each stage guided by theoretical considerations (49, 50), and to control for confounding factors such as sex. Thus, in the first stage of the hierarchical regression, we included sex as a control variable to account for its potential impact. In the second stage, we introduced smiling_sqrt_, based on preliminary evidence suggesting its relevance in medical conversations (26, 51, 52). In the final stage, we added the composite-score_sqrt_ for other facial expressions to evaluate their additional contribution to predicting communication outcomes.

**Table 2:** Results of the hierarchical regression analyses.

			**Stage**	**Predictor**	**Standardized beta**	***t***	**R^2^**	**ΔR^2^**
Quality assessment	BGR	SP	**1**	Sex (control variable)	0.22	1.59	0.048	-
			**2**	Smiling_sqrt_	−0.52	**−4.05** ^***^	**0.286** ^***^	**0.234** ^***^
			**3**	Other facial expressions_sqrt_	−0.32	**−2.58** ^*^	**0.373** ^***^	**0.087** ^*^
		Med. Student	**1**	Sex (control variable)	−0.04	−0.29	0.002	-
			**2**	Smiling_sqrt_	0.00	0.03	0.002	0.000
			**3**	Other facial expressions_sqrt_	0.09	0.58	0.008	0.006
		Com. Expert	**1**	Sex (control variable)	−0.03	−0.20	0.001	-
			**2**	Smiling_sqrt_	−0.24	−1.61	0.051	0.050
			**3**	Other facial expressions_sqrt_	0.01	0.052	0.051	0.000
	Grade	Com. Expert	**1**	Sex (control variable)	−0.01	−0.09	0.000	-
			**2**	Smiling_sqrt_	−0.33	**−2.28** ^*^	0.096^a^	**0.096** ^*^
			**3**	Other facial expressions_sqrt_	−0.058	−0.39	0.099	0.003
Level of comfort (MISS)		SP	**1**	Sex (control variable)	−0.22	−1.61	0.049	-
			**2**	Smiling_sqrt_	0.41	**3.00** ^**^	**0.197** ^**^	**0.148** ^**^
			**3**	Other facial expressions_sqrt_	−0.01	−0.09	0.197	0.000

Facial expressions (smiling and other facial expressions) served as predictors. Analyses were conducted separately for the different outcome variables (quality assessment and level of comfort). “Sex” was entered as a control variable. ^***^p < 0.001, ^**^p < 0.01, ^*^p < 0.05, - *p* > 0.1, ^a^*p* = 0.085.

#### 3.2.1 Communication quality

Controlling for sex, medical students' smiling_sqrt_ significantly predicted the BGR ratings of the SPs [*F*_(2, 49)_ = 9.83, *p* < 0.001], accounting for 28.6% of the variance. The more a medical student smiled during the consultation, the better the communication quality was rated by the SP. Introducing the composite-score_sqrt_ for the other facial expressions, further improved the predictive power (change in R^2^ being significant, *p* = 0.013) and explained an additional 8.7% of variance [*F*_(3, 48)_ = 9.53, *p* < 0.001].

Facial expressions of the medical students were not related to their own BGR (see Table 2). Neither smiling_sqrt_ [*F*_(2, 49)_ = 0.04, *p* = 0.958], nor entering the composite-score_sqrt_ for the other facial expressions did significantly explain any variation in the students' BGR ratings [*F*_(3, 48)_ = 0.14, *p* = 0.935].

Experts BGR ratings were not significantly predicted by either smiling_sqrt_ [*F*_(2, 49)_ = 1.32, *p* = 0.277], nor by entering the composite-score_sqrt_ for the other facial expressions into the model [*F*_(3, 48)_ = 0.86, *p* = 0.468] (see Table 2). In contrast, there was a trend toward a significant prediction of expert grades by smiling_sqrt_ [*F*_(2, 49)_ = 2.60, *p* = 0.085], with smiling accounting for 9.57% of variations in the grade. Entering the composite-score_sqrt_ for the other facial expressions did not significantly increase in the predictive power [*F*_(3, 48)_ = 0.153, *p* = 0.698].

In sum, medical students smiling was a better predictor of communication quality compared to the “other facial expression” category. We controlled for “sex” in all models and found that “sex” had no significant impact on communication outcomes.

#### 3.2.2 Level of comfort

Regarding the level of comfort felt by the SPs, the hierarchical multiple regression revealed that smiling_sqrt_ significantly predicted the MISS scores [*F*_(2, 49)_ = 5.99, *p* = 0.005], accounting for 19.7% of the variation in comfort level. Entering the composite-score_sqrt_ for the other facial expressions did not lead to a significant increase in explained variance [*F*_(1, 48)_ = 0.01, *p* = 0.927].

In sum, medical students smiling was a better predictor of level of comfort compared to the “other facial expression” category. Again, sex was not a significant predictor.

#### 3.2.3 Predictive power of different types of smiles (genuine/social & during listening/speaking)

Given the significant association between medical students' smiles and SPs outcome ratings, we wanted to further investigate this association by testing whether it played a role if these were genuine vs. social smiles and whether smiles were displayed while the medical students were speaking vs. listening. To this aim, linear regression analyses were performed: Smiling-while-speaking_sqrt_ or smiling-while-listening_sqrt_ and genuine-smiles_sqrt_ or social-smiles_sqrt_ served as predictors and the quality of communication or level of comfort reported by SPs served as outcomes. Again, we controlled for “sex.”

Genuine/social (see Table 3): Both genuine, as well as social smiles significantly predicted the SPs quality assessment (BGR ratings). However, genuine smiles explained significantly more variance compared to social smiles [p(Likelihood-Ratio, one-sided) = 0.021]. The SPs comfort ratings (MISS score) could also be significantly predicted by both genuine and social smiling, with neither model explaining significantly more variance in the outcome [p(Likelihood-Ratio, one-sided) = 0.112].

**Table 3:** Comparison of the effects of genuine-smile_sqrt_ vs. social-smile_sqrt_ on SPs' communication assessment.

		**Stage**	**Predictor**	**Standardized** **beta**	***t***	**R^2^**	**ΔR^2^**
Standardized patient	Quality assessment (BGR)	**1**	Sex (control variable)	0.22	1.59	0.048	-
		**2**	Genuine-smile_sqrt_	−0.46	**−3.48** ^**^	**0.237** ^**^	**0.189** ^**^
		**1**	Sex (control variable)	0.22	1.59	0.048	-
		**2**	Social-smile_sqrt_	−0.36	**−2.44** ^*^	**0.151** ^*^	**0.103** ^*^
	Level of comfort (MISS)	**1**	Sex (control variable)	−0.22	−1.61	0.049	-
		**2**	Genuine-smile_sqrt_	0.39	**2.83** ^**^	**0.183** ^**^	**0.134** ^**^
		**1**	Sex (control variable)	−0.22	−1.61	0.049	-
		**2**	Social-smile_sqrt_	0.35	**2.20** ^*^	**0.150** ^*^	**0.100** ^*^

^**^p < 0.01, ^*^p < 0.05.

Listening/speaking (see Table 4): Both smiling while speaking and listening significantly predicted the SPs quality assessment (BGR ratings). However, smiling during speaking explained significantly more variance in the outcome compared to listening [p(Likelihood-Ratio, one-sided) = 0.049]. The SPs comfort ratings (MISS score) could also be significantly predicted by both smiling during speaking, as well as during listening, with neither model explaining significantly more variance in the outcome [p(Likelihood-Ratio, one-sided) = 0.159].

**Table 4:** Comparison of the effects of smiling-while-speaking_sqrt_ vs. smiling-while-listening_sqrt_ on SPs' communication assessment.

		**Stage**	**Predictor**	**standardized** **beta**	***t***	**R^2^**	**ΔR^2^**
Standardized patient	Quality assessment (BGR)	**1**	Sex (control variable)	0.22	1.59	0.048	-
		**2**	Smiling-while-speaking_sqrt_	−0.54	**−4.39** ^***^	**0.316** ^***^	**0.268** ^***^
		**1**	Sex (control variable)	0.22	1.59	0.048	-
		**2**	Smiling-while-listening_sqrt_	−0.48	**−3.54** ^***^	**0.241** ^**^	**0.194** ^***^
	Level of comfort (MISS)	**1**	Sex (control variable)	−0.22	−1.65	0.049	-
		**2**	Smiling-while-speaking_sqrt_	0.38	**2.81** ^**^	**0.181** ^***^	**0.132** ^**^
		**1**	Sex (control variable)	−0.22	−1.65	0.049	-
		**2**	Smiling-while-listening_sqrt_	0.44	**3.18** ^**^	**0.211** ^**^	**0.162** ^**^

^***^p < 0.001, ^**^p < 0.01.

**Figure 4:**
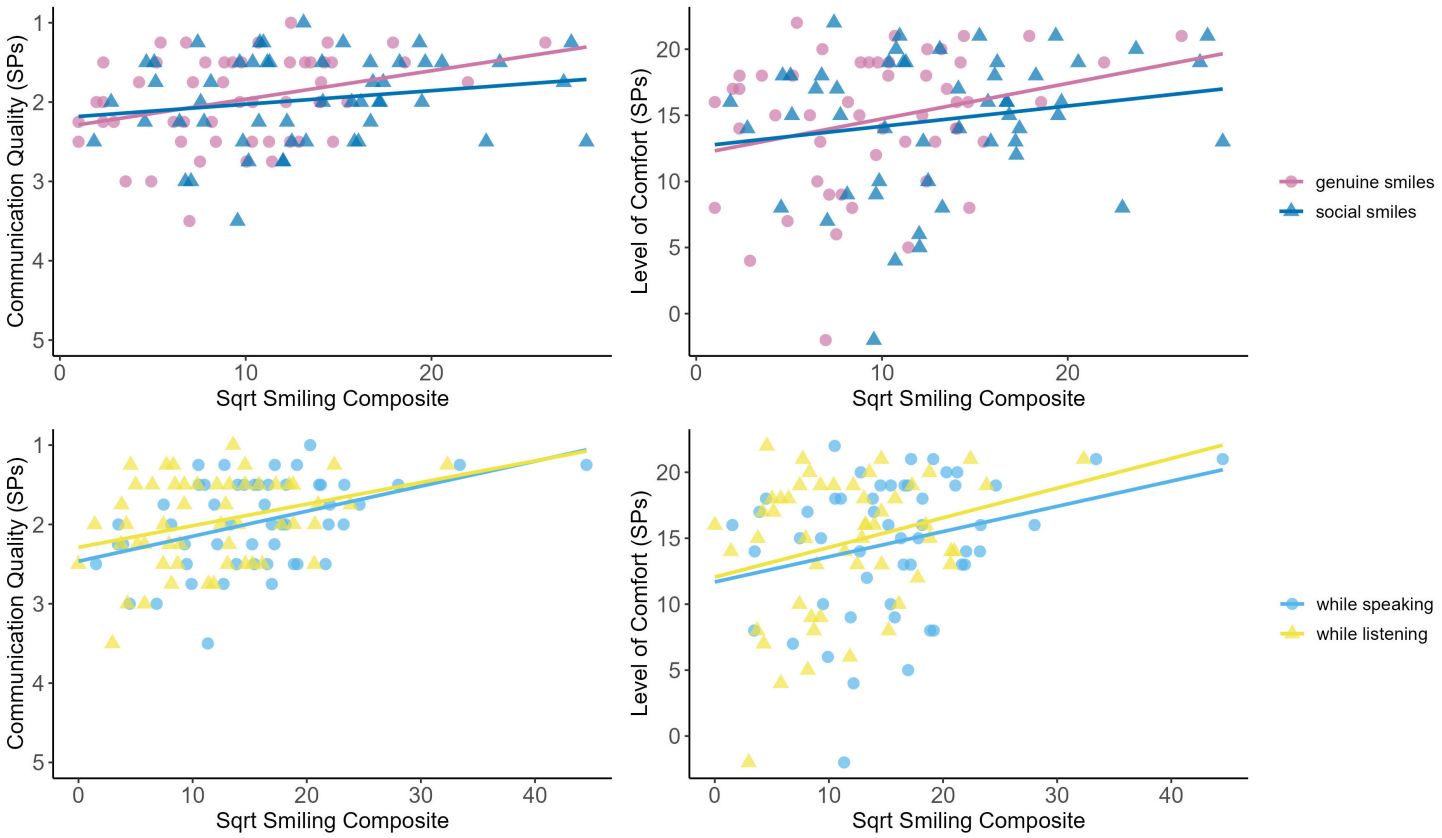
Scatterplots and regression lines showing the relationship between the different types of smiling and communication outcomes rated by the SPs. Data are presented separately for the different types of smiles (**upper panel**: genuine/social smiles; **lower panel**: during listening/speaking) as well as for communication quality (**left panel**: BGR ratings) and level of comfort (**right panel**: MISS scores) rated by the SPs. Lower ratings of communication quality indicate better performance.

In sum, genuine smiles proved to be especially powerful in predicting SPs communication assessment. In order to better visualize these findings, scatterplots depicting the associations between the different types of smiling (genuine/social & during listening/speaking) and communication outcomes (BGR ratings/MISS scores) can be found in Figure 4.

#### 3.2.4 Summary for research question (ii)

Especially smiling plays an important role in predicting the outcome of doctor-patient consultations, when considering the perspective of the SP. More frequent displays of smiling (both genuine and social) let to more positive ratings of communication quality and level of comfort. Facial expressions were less likely to predict communication outcomes assessed by the medical students themselves and by the communication experts.

## 4 Discussion

The research question we aimed to answer was two-fold, namely, to investigate (i) which facial expressions are displayed by medical students during simulated doctor-patient consultations and (ii) whether these affect the communication outcome. We found that the most prominent facial expression was “smiling,” with increased rates of smiling during speaking compared to episodes of listening. Moreover, the degree of smiling (both during listening and speaking as well as genuine and social smiles) could significantly predict communication outcomes rated by the SP.

### 4.1 Repertoire of facial expressions displayed by medical students during the consultation

We observed that the predominant category of facial expressions displayed by medical students during the simulated patient-doctor interaction was smiling, with AU 12 (Lip corner puller/zygomatic major) being the most prominent facial movement (on average displayed for almost 2 min during the 7-min simulated consultation). Notably, about 50% of zygomatic major activity (AU 12) coincided with orbicularis oculi activity (orbit tightening, AU 06 and/or AU 07). According to Ekman et al. (29, 46) this combination is regarded as indicative of a sincere or authentic smile (Duchenne smile). Conversely, zygomatic major activity alone (non-Duchenne smile) (45) is typically associated with smiles more consciously generated for social reasons (47, 53). Thus, our finding suggests that half of the smiles displayed by medical students may be socially motivated and occurred with similar frequencies while speaking (55%) and while listening (46%).

Smiles (regardless of being accompanied by orbicularis oculi activity) are associated with compassion, empathy, and friendliness (51). Furthermore, they play a crucial role in fostering trust, as individuals tend to place greater trust in those who smile compared to those who do not (54) and thus, smiles can serve as a social bonding agent or a social glue (55, 56). Additionally, the act of smiling can initiate emotional contagion, creating a shared sense of happiness between both parties (57). Consequently, the smiles of medical students in the simulated medical consultations may indicate their inclination to establish a reliable and positive patient-doctor relationship.

To further understand the nuances of medical students' smiles, we compared the frequency of facial expressions when medical students were speaking vs. listening. Medical students exhibited significantly more smiles when they were actively speaking compared to listening. It is possible that medical students accompanied their verbal expressions by smiling as means of conveying warmth, empathy, and a positive and supportive communication environment. Moreover, when they were listening, they may have been more focused on processing information and understanding the SPs. Thus, the act of listening might be encoded in a different set of non-verbal cues. Indeed, we found that besides smiling, medical students also displayed other AUs (AUs 01, 02, 04, 25, 26). Even though it is challenging to attribute specific meaning or affect labels to these AUs, previous findings suggest that AUs 01, 02, and 04 are linked to interest (58, 59) or attentive listening. Therefore, these facial expressions might potentially also serve as important non-verbal cues contributing to the establishment of a trustworthy relationship.

### 4.2 Prediction of communication outcomes

When investigating the power of facial expressions to predict communication outcomes, we found notable variations depending on who was evaluating the communication. This is probably due to the diverse perspectives held by SPs, medical students, and communication experts in evaluating the medical consultations. As has been previously shown, each evaluator holds a unique viewpoint that influences their assessment (66), for example SPs have been found to be less strict when rating students' performance (60).

*Communication evaluated by SPs:* We identified a strong association between the facial expressions of medical students and SPs evaluation of the communication quality. Both smiling and other facial expressions significantly contributed to the regression model, explaining together approximately 37% of the variance in communication quality. This suggests that the more a medical student displayed smiling along with other facial expressions, the better was the communication quality rated by the SP. This finding fits well with a previous study that used photos of physicians that differed with regard to the degree of smiling and found that physicians who are smiling were rated as being more competent (25, 26). Regarding SPs level of comfort, medical students' smiling significantly predicted SPs' level of comfort, whereas other facial expressions did not have an additional impact. The significant association between smiling and SPs level of comfort aligns with the notion that a warm, welcoming, and trustful demeanor, as conveyed through smiling, can positively influence patient perceptions (26, 51, 52). Moreover, the significance of smiling in influencing patients level of comfort is supported by Mast et al. (24) study, where the frequency of smiling (smiles were simply counted by observers, with no in-depth facial analyses) predicted patient satisfaction. Similarly, Wong et al. (27) demonstrated that hindering patients from observing smiles due to doctors wearing face masks had a significant negative effect on perceptions of empathy. Notably, our study differs from these previous studies as the first to systematically code smiling using the Facial Action Coding System (FACS); which allows for a nuanced, reliable and valid analysis of facial expression (35).

It is important to note that smiling can be interpreted differently by the recipient based on the situational context. For instance, while an intensive, genuine smile during a greeting may be perceived as welcoming, the same smile during a patient's report of their concerns may be considered inappropriate. Therefore, we analyzed whether the type of smile (genuine vs. social) and whether the smile was displayed during speaking or listening had differential effects on SPs assessment of the communication. We found that medical students' smiling during both speaking and listening as well as genuine and social smiles all significantly predicted communication quality and the level of comfort.

*Communication evaluated by medical students:* Neither smiling nor the other facial expressions significantly predicted how medical students rated their own communication performance. This result is unexpected, as one might assume that smiling is linked to feeling more comfortable and secure during interaction, or that smiling leads to more confidence due to facial feedback mechanisms (61, 62). However, the effects of facial feedback are small, short lasting (63) and heterogeneous, potentially having a greater impact on emotional experience than on structured assessment.

*Communication evaluated by experts:* While facial expressions of medical students could not predict experts' evaluation of the communication quality using the BGR, smiling significantly predicted the grade assigned to the communication performance. Nonetheless, the degree of explained variance was also relatively small. Communication experts watched video recordings capturing the whole body of the medical student and the SPs and thus, were less able to perceive facial expressions but focused more on formal aspects of the communication. Therefore, it may not be surprising that we did not observe strong effects of the medical students' facial expressions on the nuanced quality assessment by the communication experts.

### 4.3 Limitations

While simulated doctor-patient interactions allowed for controlled assessments, it may not fully capture real-world patient experiences and clinical complexities. As such, the applicability of these results to actual clinical settings may vary, as real patients' needs and expectations can differ significantly. While smiling may enhance patient comfort in some clinical situations, it might be less suitable in more challenging or serious clinical discussions. Moreover, although we found that SPs perceived higher conversation quality and comfort when medical students displayed more smiles, this does not necessarily guarantee that the conversation was objectively effective, e.g., in terms of patient comprehension or adherence to treatment recommendations. As we conducted our study with SPs, we could not assess whether patients comprehended medical information effectively. Therefore, it remains unknown whether the improved perceived conversation quality correlated with better patient understanding and decision-making. Furthermore, our study is limited regarding sample size and sample diversity, which might limit the generalizability of our findings. With 40 women and only 12 men we have a highly unbalanced sex distribution in our sample, which may have affected the power to detect potential sex differences. Thus, we cannot exclude that our findings mainly apply to female doctors. Given that sex differences in smiling have been found, with females smiling more frequently especially in social settings (64) and females' smiles being perceived as more genuine and trustworthy compared to males' (65) it is possible, that smiling might be less relevant or less advantageous for male doctors. Moreover, our sample was too small to explore potential differences depending on whether medical students and SPs were of the same or different sex, which might also have impacted smiling patterns (56). Furthermore, we only focused on facial expressions of the medical students due to the enormous time it takes to FACS-code the data (20 h to code one medical student). It is crucial to acknowledge, however, that facial expressions are a result of a dynamic interaction between doctor and patient and are perceived simultaneously alongside other verbal and non-verbal cues. By isolating only facial expressions of the medical students, we cannot capture the complex interplay of doctor-patient interactions.

## 5 Conclusions

We have shown that (i) smiling is the most frequent facial expression displayed by medical students in a simulated doctor-patient interaction. Reasons for this frequent display of smiling might be to convey warmth, empathy, and a positive and supportive communication environment for the patient. Along this notion, we could also show (ii) that smiling significantly predicted how the SPs rated the interaction. Future studies should try to assess facial expressions in real clinical settings to see whether smiling is also the predominant emotional facial expression displayed by doctors or whether the type of facial expression depends on the clinical setting and type of consultation or type of medical procedure. Smiling might be effective and be appropriate only in certain clinical settings.

Our findings suggest that smiling can be an efficient method for clinicians to build a positive and comfortable environment for their patients. Interestingly, it seems to be of little impact whether these smiles are displayed during speaking or during listening or whether these smiles represent genuine or social smiles to elicit these positive effects. Thus, medical training programs should highlight the impact of positive facial expressions on patient outcomes and use role-playing exercises with SPs to learn how to use “smiling” appropriately in different clinical settings (e.g., while smiling might be positive in some settings, it must be balanced in more serious discussions, such as delivering bad news).

## Data Availability

The original contributions presented in the study are included in the article/Supplementary material, further inquiries can be directed to the corresponding author.

## References

[1] Simpson M Buckman R Stewart M Maguire P Lipkin M Novack D. Doctor-patient communication: the Toronto consensus statement. BMJ. (1991) 303:1385. 10.1136/bmj.303.6814.1385 1760608 PMC1671610

[2] Makoul G Schofield T. Communication teaching and assessment in medical education: an international consensus statement. Patient Educ Couns. (1999) 37:191–5. 10.1016/S0738-3991(99)00023-3 14528545

[3] Bachmann C Abramovitch H Barbu G Cavaco MA Elorza RD Haak R. A European consensus on learning objectives for a core communication curriculum in health care professions. Patient Educ Couns. (2013) 93:18–26. 10.1016/j.pec.2012.10.016 23199592

[4] Robinson JD. Nonverbal communication and physician-patient interaction: review and new directions. In:Manusov VL Patterson ML., editors *The SAGE handbook of nonverbal communication*. Thousand Oaks, CA: Sage Publication (2006). p. 437–460. 10.4135/9781412976152.n23

[5] Schmid Mast M. On the importance of nonverbal communication in the physician–patient interaction. Patient Educ Couns. (2007) 67:315–8. 10.1016/j.pec.2007.03.005 17478072

[6] Kerse N Buetow S Mainous AG Young G Coster G Arroll B. Physician-patient relationship and medication compliance: a primary care investigation. Ann Fam Med. (2004) 2:455–61. 10.1370/afm.139 15506581 PMC1466710

[7] Ridd M Shaw A Lewis G Salisbury C. The patient-doctor relationship: a synthesis of the qualitative literature on patients' perspectives. Br J General Pract. (2009) 59:e116–33. 10.3399/bjgp09X420248 19341547 PMC2662123

[8] Del Canale S Louis DZ Maio V Wang X Rossi G Hojat M. The relationship between physician empathy and disease complications: an empirical study of primary care physicians and their diabetic patients in Parma, Italy. Acad Med. (2012) 87:1243–9. 10.1097/ACM.0b013e3182628fbf 22836852

[9] Kelley JM Kraft-Todd G Schapira L Kossowsky J Riess H. The influence of the patient-clinician relationship on healthcare outcomes: a systematic review and meta-analysis of randomized controlled trials. PLoS ONE. (2014) 9:e94207. 10.1371/journal.pone.0094207 24718585 PMC3981763

[10] Deveugele M Derese A Maesschalck S de Willems S van Driel M. Teaching communication skills to medical students, a challenge in the curriculum? Patient Educ Couns. (2005) 58:265–70. 10.1016/j.pec.2005.06.004 16023822

[11] Bachmann C Barzel A Roschlaub S Ehrhardt M Scherer M. Can a brief two-hour interdisciplinary communication skills training be successful in undergraduate medical education? Patient Educ Couns. (2013) 93:298–305. 10.1016/j.pec.2013.05.019 23806818

[12] Chant S Tim RJ Russell G Webb C. Communication skills training in healthcare: a review of the literature. Nurse Educ Today. (2002) 22:189–202. 10.1054/nedt.2001.0690 12027600

[13] Kienle R Freytag J Lück S Eberz P Langenbeck S Sehy V. Communication skills training in undergraduate medical education at Charité – Universitätsmedizin Berlin. GMS J Med Educ. (2021) 38. 10.3205/zma001452 33824892 PMC7994867

[14] Kiessling C Dieterich A Fabry G Hölzer H Langewitz W Mühlinghaus I. Communication and social competencies in medical education in German-speaking countries: the Basel Consensus Statement. Results of a Delphi Survey. Patient Educ Counsel. (2010) 81:259–66. 10.1016/j.pec.2010.01.017 20223614

[15] Hall JA Irish JT Roter DL Ehrlich CM Miller LH. Satisfaction, gender, and communication in medical visits. Med Care. (1994) 32:1216–31. 10.1097/00005650-199412000-00005 7967860

[16] Hall JA Harrigan JA Rosenthal R. Nonverbal behavior in clinician—patient interaction. Appl Prev Psychol. (1995) 4:21–37. 10.1016/S0962-1849(05)80049-6

[17] Griffith CH Wilson JF Langer S Haist SA. House staff nonverbal communication skills and standardized patient satisfaction. J Gen Intern Med. (2003) 18:170–4. 10.1046/j.1525-1497.2003.10506.x 12648247 PMC1494838

[18] Ambady N Koo J Rosenthal R Winograd CH. Physical therapists' nonverbal communication predicts geriatric patients' health outcomes. Psychol Aging. (2002) 17:443–52. 10.1037/0882-7974.17.3.443 12243386

[19] Argyle M. Bodily Communication. 2^nd^ ed. London, New York: Routledge (1988).

[20] Riess H Kraft-Todd G. EMPATHY a tool to enhance nonverbal communication between clinicians and their patients. Academic Med. (2014) 89:1108–1112. 10.1097/ACM.0000000000000287 24826853

[21] Zhang X Li L Zhang Q Le LH Wu Y. Physician empathy in doctor-patient communication: a systematic review. Health Commun. (2024) 39:1027–37. 10.1080/10410236.2023.2201735 37062918

[22] Harrigan JA Oxman TE Rosenthal R. Rapport expressed through nonverbal behavior. J Nonverbal Behav. (1985) 9:95–110. 10.1007/BF00987141

[23] Kraft-Todd GT Reinero DA Kelley JM Heberlein AS Baer L Riess H. Empathic nonverbal behavior increases ratings of both warmth and competence in a medical context. PLoS ONE. (2017) 12:e0177758. 10.1371/journal.pone.0177758 28505180 PMC5432110

[24] Mast MS Hall JA Klöckner C Choi E. Physician gender affects how physician nonverbal behavior is related to patient satisfaction. Med Care. (2008) 46:1212–8. 10.1097/MLR.0b013e31817e1877 19300310

[25] Hall JA Ruben MA Swatantra Swatantra First impressions of physicians according to their physical and social group characteristics. J Nonverbal Behav. (2020) 44:279–299. 10.1007/s10919-019-00329-8

[26] Epstein N Brendel T Hege I Ouellette DL Schmidmaier R Kiesewetter J. The power of the pen: how to make physicians more friendly and patients more attractive. Med Educ. (2016) 50:1214–8. 10.1111/medu.13002 27873423

[27] Wong CKM Yip BHK Mercer S Griffiths S Kung K Wong MC. Effect of facemasks on empathy and relational continuity: a randomized controlled trial in primary care. BMC Fam Pract. (2013) 14:200. 10.1186/1471-2296-14-200 24364989 PMC3879648

[28] Park KH Park SG. The effect of communication training using standardized patients on nonverbal behaviors in medical students. Korean J Med Educ. (2018) 30:153–9. 10.3946/kjme.2018.90 29860781 PMC5990900

[29] Ekman P Friesen WV Hager JC. Facial action coding system: Investigator‘s guide. Salt Lake City, Utah: Research Nexus (2002).

[30] Ekman P. Methods for measuring facial action. In: Handbook of Methods in Nonverbal Behavior Research (1982). p. 45–90.

[31] Izard CE. Maximally Discriminative Facial Movement Coding System. Newark: University of Delaware (1979).

[32] Cohn J Kanade T Moriyama T Ambadar Z Xiao J Gao J. A comparative study of alternative FACS coding algorithms. Technical Report, Robotics Institute, Carnegie Mellon University. (2001).

[33] Faul F Erdfelder E Lang A-G Buchner A. G^*^Power 3: a flexible statistical power analysis program for the social, behavioral, and biomedical sciences. Behav Res Methods. (2007) 39:175–91. 10.3758/BF03193146 17695343

[34] Cohn J Ambadar Z Ekman P. Observer-based measurement of facial expression with the facial action coding system. In:Coan JA Allen JJB., editors. Handbook of Emotion Elicitation and Assessment. New York, NY, United States: Oxford University Press (2007). p. 203–221. 10.1093/oso/9780195169157.001.0001

[35] Ekman P Friesen WV. The Facial Action Coding System (FACS): A technique for the measurement of Tacial Action. Palo Alto, CA: Consulting Psychologists Press (1978). 10.1037/t27734-000

[36] Hodges B McIlroy JH. Analytic global OSCE ratings are sensitive to level of training. Med Educ. (2003) 37:1012–6. 10.1046/j.1365-2923.2003.01674.x 14629415

[37] Scheffer S. “Validierung des „Berliner Global Rating“ (BGR) - ein Instrument zur Prüfung kommunikativer Kompetenzen Medizinstudierender im Rahmen klinisch-praktischer Prüfungen (OSCE) [Validation of the “Berlin Global Rating” (BGR) - an instrument for assessing the communicative competencies of medical students in clinical-practical examinations (OSCE)],”. Dissertation, Charité - Universitätsmedizin Berlin. (2009).

[38] Zimmermann A Baerwald C Fuchs M Girbardt C Götze H. The Longitudinal Communication Curriculum at Leipzig University, Medical Faculty – implementation and first experiences. GMS J Med Educ. (2021) 38:Doc58. 10.3205/zma001454 33824894 PMC7994878

[39] Spanke J Raus C Haase A Angelow A Ludwig F Weckmann G. Fairness and objectivity of a multiple scenario objective structured clinical examination. GMS J Med Educ. (2019) 36:Doc26. 10.3205/zma001234 31211221 PMC6545613

[40] Koo TK Li MY. A guideline of selecting and reporting intraclass correlation coefficients for reliability research. J Chiropr Med. (2016) 15:155–63. 10.1016/j.jcm.2016.02.012 27330520 PMC4913118

[41] Meakin R Weinman J. The ‘Medical Interview Satisfaction Scale' (MISS-21) adapted for British general practice. Fam Pract. (2002) 19:257–63. 10.1093/fampra/19.3.257 11978716

[42] Balestrieri M Girolamo G de Rucci P. Construct validity and psychosocial correlates of the Italian version of the 21-item Medical Interview Satisfaction Scale in primary care. BJPsych Open. (2021) 7:e57. 10.1192/bjo.2020.164 33597072 PMC8058927

[43] Kunz M Meixner D Lautenbacher S. Facial muscle movements encoding pain-a systematic review. Pain. (2019) 160:535–49. 10.1097/j.pain.0000000000001424 30335682

[44] Ekman P. Should we call it expression or communication? Innovation. (1997) 10:333–44. 10.1080/13511610.1997.9968538

[45] Ekman P Friesen WV. Felt, false, and miserable smiles. J Nonverbal Behav. (1982) 6:238–52. 10.1007/BF00987191

[46] Frank MG Ekman P. Not all smiles are created equal: the differences between enjoyment and nonenjoyment smiles. Humr. (1993) 6:9–26. 10.1515/humr.1993.6.1.9

[47] Ekman P Davidson RJ Friesen WV. The Duchenne smile: Emotional expression and brain physiology: II. J Pers Soc Psychol. (1990) 58:342–53. 10.1037/0022-3514.58.2.342 2319446

[48] Osborne J. Notes on the use of data transformations. Pract Assess Res Evalu. (2019) 8:6. 10.7275/4vng-5608

[49] Flom L Cassell D. Stopping stepwise: why stepwise and similar selection methods are bad, and what you should use. In: NorthEast SAS Users Group Inc 20^th^ Annual Conference: 11-14^th^ November 2007. Baltimore, Maryland (2007).

[50] Petrocelli JV. Hierarchical multiple regression in counseling research: common problems and possible remedies. Measur Eval Couns Dev. (2003) 36:9–22. 10.1080/07481756.2003.12069076

[51] Beamish AJ Foster JJ Edwards H Olbers T. What's in a smile? A review of the benefits of the clinician's smile. Postgrad Med J. (2019) 95:91–5. 10.1136/postgradmedj-2018-136286 30700580

[52] Laughey W Sangvik Grandal N M Finn G. Medical communication: the views of simulated patients. Med Educ. (2018) 52:664–676. 10.1111/medu.13547 29600570

[53] Fox NA Davidson RJ. Patterns of brain electrical activity during facial signs of emotion in 10-month-old infants. Dev Psychol. (1988) 24:230–6. 10.1037/0012-1649.24.2.230

[54] Scharlemann JP Eckel CC Kacelnik A Wilson RK. The value of a smile: game theory with a human face. J Econ Psychol. (2001) 22:617–40. 10.1016/S0167-4870(01)00059-9

[55] Suzuki K Yokoyama M Yoshida S Mochizuki T Yamada T Narumi T. Faceshare: Mirroring with pseudo-smile enriches video chat communications. In: Proceedings of the 2017 CHI Conference on Human Factors in Computing Systems (2017). p. 5313–5317. 10.1145/3025453.3025574

[56] Hess U Bourgeois P. You smile–I smile: emotion expression in social interaction. Biol Psychol. (2010) 84:514–20. 10.1016/j.biopsycho.2009.11.001 19913071

[57] Dimberg U Thunberg M. Empathy, emotional contagion, and rapid facial reactions to angry and happy facial expressions. PsyCh J. (2012) 1:118–27. 10.1002/pchj.4 26272762

[58] Campos B Shiota MN Keltner D Gonzaga GC Goetz JL. What is shared, what is different? Core relational themes and expressive displays of eight positive emotions. Cogn Emot. (2013) 27:37–52. 10.1080/02699931.2012.683852 22716231

[59] Babaei E Srivastava N Newn J Zhou Q Dingler T Velloso E. Faces of focus: a study on the facial cues of attentional states. In: Proceedings of the 2020 CHI Conference on Human Factors in Computing Systems. New York, NY, United States: Association for Computing Machinery (2020). p. 1–13. 10.1145/3313831.3376566

[60] Heine N Garman K Wallace P Bartos R Richards A. An analysis of standardised patient checklist errors and their effect on student scores. Med Educ. (2003) 37:99–104. 10.1046/j.1365-2923.2003.01416.x 12558879

[61] Soussignan R. Duchenne smile, emotional experience, and autonomic reactivity: a test of the facial feedback hypothesis. Emotion. (2002) 2:52–74. 10.1037/1528-3542.2.1.52 12899366

[62] Coles NA Larsen JT Lench HC. A meta-analysis of the facial feedback literature: Effects of facial feedback on emotional experience are small and variable. Psychol Bull. (2019) 145:610–51. 10.1037/bul0000194 30973236

[63] Söderkvist S Ohlén K Dimberg U. How the experience of emotion is modulated by facial feedback. J Nonverbal Behav. (2018) 42:129–51. 10.1007/s10919-017-0264-1 29497224 PMC5816132

[64] LaFrance M Hecht MA Paluck EL. The contingent smile: a meta-analysis of sex differences in smiling. Psychol Bull. (2003) 129:305–34. 10.1037/0033-2909.129.2.305 12696842

[65] Galinsky DF Erol E Atanasova K Bohus M Krause-Utz A Lis S. Do I trust you when you smile? Effects of sex and emotional expression on facial trustworthiness appraisal. PLoS ONE. (2020) 15:e0243230. 10.1371/journal.pone.0243230 33270729 PMC7714177

[66] Gordon HS Street RL. How physicians, patients, and observers compare on the use of qualitative and quantitative measures of physician-patient communication. Eval Health Prof. (2016) 39:496–511. 10.1177/0163278715625737 26755527 PMC4939284

