# Supplementary material for: Smiling doctor, satisfied patient—the impact of facial expressions on doctor-patient interactions
Source: Front Med (Lausanne). 2025 Apr 24;12:1518517. doi: 10.3389/fmed.2025.1518517 (PMC12058742; doi:10.3389/fmed.2025.1518517)
Supplement: Supplementary file 2 [file Table_1.docx]

Supplementary Material

**Table S1**: All Action Units (AUs) being displayed by the medical students during the 7 minutes of consultation. The AUs are ordered based on their duration.

| **Action Unit** | **Description** | **Muscle** | **Mean duration (in seconds) ± SD** | **% of occurrence** |
| --- | --- | --- | --- | --- |
| AU50_doctor | Doctor speaking | -- | 219.67 ± 47.88 | 52.0 |
| AU50_patient | Patient speakting | -- | 198.06 ± 33.19 | 46.9 |
| AU_12* | Lip Corner Puller | zygomatic major | 116.13 ± 98.09 | 27.5 |
| AU_7* | Lid Tightener | orbicularis oculi | 85.05 ± 74.86 | 20.2 |
| AU_25* | Lips Part | orbicularis oris | 45.19 ± 31.81 | 10.7 |
| AU_26* | Jaw Drop | orbicularis oris | 35.02 ± 27.04 | 8.3 |
| AU_6* | Cheek Raiser and Lid Compressor | orbicularis oculi | 28.30 ± 64.30 | 6.7 |
| AU_1* | Inner Brow Raiser | frontalis | 22.04 ± 33.03 | 5.2 |
| AU_2* | Outer Brow Raiser | frontalis | 20.48 ± 30.95 | 4.9 |
| AU_4* | Brow Lowerer | corrugator supercilli | 12.09 ± 17.59 | 2.9 |
| AU_10 | Upper Lip Raiser | levator labii superioris, caput infraorbitalis | 9.73 ± 21.35 | 2.3 |
| AU_30 | Jaw Sideways |  | 6.40 ± 8.01 | 1.5 |
| AU_15 | Lip Corner Depressor | depressor anguli oris | 5.13 ± 6.82 | 1.2 |
| AU_28 | Lips Suck | orbicularis oris | 5.12 ± 4.72 | 1.2 |
| AU_14 | Dimpler | buccinator | 4.60 ± 5.49 | 1.1 |
| AU_17 | Chin Raiser | mentalis | 3.91 ± 4.17 | 0.9 |
| AU_5 | Upper Lid Raiser | levator palpebrae superioris, superior tarsal muscle | 3.44 ± 5.00 | 0.8 |
| AU_20 | Lip Strecher | risorius with platysma | 3.25 ± 5.03 | 0.8 |
| AU_24 | Lip Presser | orbicularis oris | 3.24 ± 3.78 | 0.8 |
| AU_23 | Lip Tightener | orbicularis oris | 2.87 ± 2.81 | 0.7 |
| AU_18 | Lip Pucker | incisivii labii superioris and incisivii labii inferioris | 2.70 ± 3.78 | 0.6 |
| AU_32 | Bite |  | 2.47 ± 2.73 | 0.6 |
| AU_29 | Jaw Thrust |  | 2.39 ± 1.82 | 0.6 |
| AU_19 | Tongue Show |  | 1.94 ± 1.62 | 0.5 |
| AU_16 | Lower Lip Depressor | depressor labii inferioris | 1.61 ± 1.53 | 0.4 |
| AU_36 | Bulge |  | 1.40 ± 0.71 | 0.3 |
| AU_37 | Lip Wipe |  | 1.39 ± 1.35 | 0.3 |
| AU_27 | Mouth Stretch | pterygoids, digastric | 1.14 ± 0.65 | 0.3 |
| AU_8 | Lips Toward Each Other | orbicularis oris | 1.02 ± 0.88 | 0.2 |
| AU_22 | Lip Funneler | orbicularis oris | 0.59 ± 0.28 | 0.1 |
| AU_35 | Suck |  | 0.53 ± 0.47 | 0.1 |
| AU_9 | Nose Wrinkler | levator labii superioris alaeque nasi | 0.43 ± 0.09 | 0.1 |

**AUs were displayed on average >10 seconds during the 7 minutes consultation and were used for further analyses*
